# Minimum toe clearance events in divided attention treadmill walking in older and young adults: a cross-sectional study

**DOI:** 10.1186/s12984-015-0052-2

**Published:** 2015-07-12

**Authors:** Braveena K. Santhiranayagam, Daniel T. H. Lai, W. A. Sparrow, Rezaul K. Begg

**Affiliations:** Institute of Sport, Exercise and Active Living (ISEAL), Victoria University, PO Box 14428, Melbourne, Victoria 8001 Australia; College of Sport & Exercise Science, Victoria University, PO Box 14428, Melbourne, Victoria 8001 Australia; College of Engineering & Science, Victoria University, PO Box 14428, Melbourne, Victoria 8001 Australia

**Keywords:** Gait, Minimum Toe Clearance (MTC), MTC_Height, MTC_Time, Attention division, Dual task walking, Non-MTC gait cycles

## Abstract

**Background:**

Falls in older adults during walking frequently occur while performing a concurrent task; that is, dividing attention to respond to other demands in the environment. A particularly hazardous fall-related event is tripping due to toe-ground contact during the swing phase of the gait cycle. The aim of this experiment was to determine the effects of divided attention on tripping risk by investigating the gait cycle event Minimum Toe Clearance (MTC).

**Methods:**

Fifteen older adults (mean 73.1 years) and 15 young controls (mean 26.1 years) performed three walking tasks on motorized treadmill: (i) at preferred walking speed (preferred walking), (ii) while carrying a glass of water at a comfortable walking speed (dual task walking), and (iii) speed-matched control walking without the glass of water (control walking). Position-time coordinates of the toe were acquired using a 3 dimensional motion capture system (Optotrak NDI, Canada). When MTC was present, toe height at MTC (MTC_Height) and MTC timing (MTC_Time) were calculated. The proportion of non-MTC gait cycles was computed and for non-MTC gait cycles, toe-height was extracted at the mean MTC_Time.

**Results:**

Both groups maintained mean MTC_Height across all three conditions. Despite greater MTC_Height SD in preferred gait, the older group reduced their variability to match the young group in dual task walking. Compared to preferred speed walking, both groups attained MTC earlier in dual task and control conditions. The older group’s MTC_Time SD was greater across all conditions; in dual task walking, however, they approximated the young group’s SD. Non-MTC gait cycles were more frequent in the older group across walking conditions (for example, in preferred walking: young – 2.9 %; older - 18.7 %).

**Conclusions:**

In response to increased attention demands older adults preserve MTC_Height but exercise greater control of the critical MTC event by reducing variability in both MTC_Height and MTC_Time. A further adaptive locomotor control strategy to reduce the likelihood of toe-ground contacts is to attain higher mid-swing clearance by eliminating the MTC event, i.e. demonstrating non-MTC gaits cycles.

## Background

Unintentional falls, the primary cause of accidental injury and falls rates in older people are estimated to be 30–40 % annually [1]. In the United States and other developed countries, falls are so prevalent as to be one of the leading causes of death in people aged over 65 years [2]. In efforts to identify the causes of falling, an interaction of extrinsic and intrinsic risk factors has been recognised. Extrinsic factors are typically environmental features such as uneven or raised surfaces and ground-based obstacles. Intrinsic factors include sensorimotor deficits, cognitive declines and perceptual impairments, some of which are due to ageing. From a biomechanical perspective the three most frequent direct causes of falling are tripping, slipping and balance loss [3, 4]. Of these, tripping accounts for more than 50 % of falls [4] and in community-dwelling older adults, there is a high association between tripping frequency and falling [5]. Recent falls monitoring of frail older adults in long-term residential care facilities showed, for example that 49 % of falls occurred while walking and 21 % were caused by tripping [6]. An experimental study by Sparrow et al. [7] found increased attention demands for the older group and greater attention cost for both young and older groups in more challenging foot-targeting task. Combining these experimental findings and reports that the majority of falls during walking occur while dividing attention [8–10], it was considered important to conduct a controlled experiment to discover how older adults adapt their gait while dividing attention.

Tripping results directly from unsuccessful toe-ground clearance, primarily during the swing phase of a gait cycle. Previous research with both young and older populations have, therefore, focused on how lower limb swing-phase trajectory control influences toe-ground clearance, represented by a biomechanical event during the mid-swing phase of the gait cycle, Minimum Toe Clearance (MTC) [11–13]. MTC is a critical representation of toe-trajectory control related to locomotion due to its low (~10-20 mm) toe-ground clearance (MTC_Height), high foot velocity (~4.60 m/s) and a single-foot base of support. Failure to adequately negotiate surface height variability by adjusting clearance at MTC can increase the risk of tripping. To maintain the clearance, older adults demonstrate similar mean toe-ground height at MTC (MTC_Height) as young individuals [12, 13]. In contrast, the MTC_Height distribution, characterized using either the standard deviation (SD) or inter quartile range (IQR), increases with ageing and this greater stride-to-stride variability in MTC_Height appears to increase tripping risk [12–14]. Nordin et al. [15] however, reported that older individuals aged 75 years and above who demonstrated change in gait parameters such as mean step-width, mean step-time and step-length variability when walking while carrying a cup and saucer were less falls prone. In answer to the question of how divided attention during walking changes MTC_Height, recent work by Schulz et al. [16] with young adults showed *no difference* in mean MTC_Height during walking in a divided attention condition in which a glass of water was carried on a tray. While their experiment provided useful background to divided attention effects on MTC, they neither reported MTC_Height variability nor included older adults in the experimental design. Further, MTC as an *event* has two characteristics, not only MTC_Height, as discussed above but also timing of MTC event, i.e., time of MTC event occurrence within the gait cycle (“MTC_Time”). While MTC_Height has been investigated extensively [14] MTC_Time has been less frequently discussed [13].

The MTC literature reviewed above led to the following research question; how would age and divided attention influence MTC_Time in addition to MTC_Height? Given earlier observations [16] that younger people are not hampered by dual task constraints the younger individuals were not expected to require adaptations to MTC_Time in addition to MTC_Height. In contrast, it was hypothesised that to reduce the likelihood of ground contact older participants would increase MTC_Height, reduce mean MTC_Time and reduce MTC event variability (both MTC_Height and MTC_Time) in the more attention demanding dual task condition. Given the absence of previous research findings on MTC timing characteristics, the above hypothesised MTC_Time related age and condition effects were more speculative than for MTC_Height. From a biomechanical perspective, however, by attaining MTC earlier (shorter MTC_Time), it was expected that the walker may transit more quickly from the hazardous low-clearance zone of the toe trajectory to the higher clearance phase, possibly reducing tripping risk.

Furthermore, a study of obstacle effects on MTC in young adults [17] revealed, however, that only approximately 98 % of gait cycles in unconstrained preferred speed walking, and 80 % during obstacle crossing did demonstrate gait cycles with an MTC event. These results implied that some gait cycles do *not* demonstrate an MTC event, but such gait cycles have not previously been measured in young and older adults. It was also of interest to further investigate the gait cycles which do not show an MTC event in both age groups, by comparing preferred walking with a divided attention task that did not require changes to lower limb trajectory due to significant obstacles. To minimise the possibility of toe-ground contact at the MTC event it was hypothesised that older adults would exhibit a greater proportion of gait cycles without an MTC event.

## Methods

### Participants

The experiment was carried out in the Victoria University Biomechanics Laboratory, Melbourne, Australia. Based on data presented in previous MTC studies comparing young and older adults [13, 18] samples of 15 young and 15 older adults (Table 1) were considered sufficient to demonstrate any group and condition effects on the biomechanical variables. As per the responses to a health questionnaire, older individuals with the ability to perform everyday walking for 30 min without a walking aid and having no orthopaedic, respiratory and cardiac conditions were recruited. They also underwent following screening tests: (i) timed up and go (<13.5 s [18]), (ii) visual acuity (>6/12) and (iii) contrast sensitivity (Melbourne edge test > 6/15 [19]).

**Table 1:** Young and older participants’ physical characteristics, preferred walking speed and dual task walking speed

Variable	Young (n = 15)	Old (n = 15)	*p* value
	Mean (SD)	Mean (SD)	
Age (years)	26.1 (3.8)	73.1 (5.6)	<10^−3^*
Body mass (kg)	72.4 (7.6)	71.5 (15.2)	0.848
Stature (m)	175.1 (7.9)	167.9 (9.2)	0.014*
Preferred walking speed (m/s)	1.06 (0.14)	0.94 (0.42)	0.067
Dual Task walking speed (m/s)	0.53 (0.09)	0.42 (0.08)	<10^−3^*
Gender	4 F, 11 M	7 F, 8 M	-

F = Female, M = Male, *= p < 0.05

### Experimental protocol

**Fig. 1:**
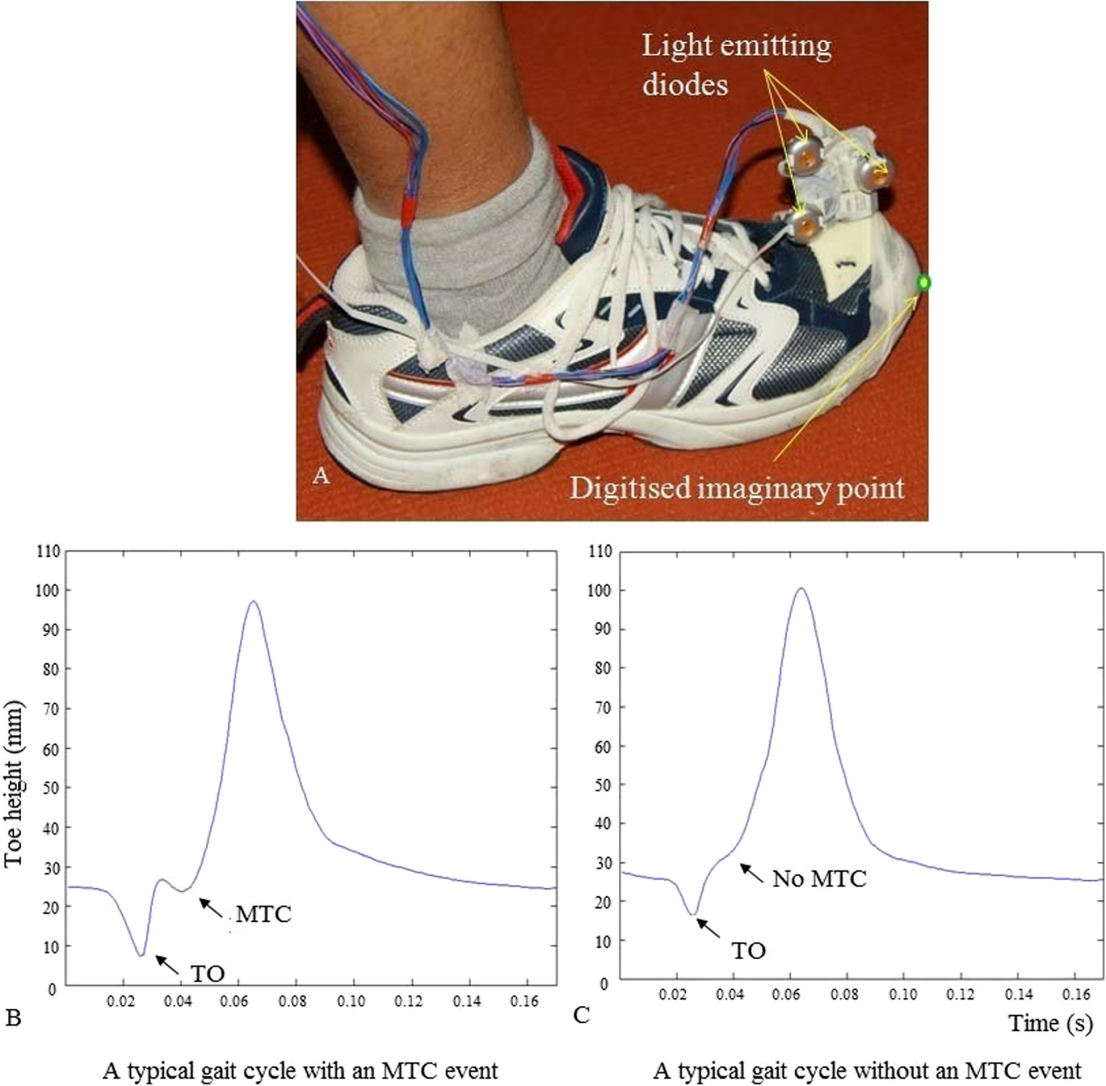
**a** Rigid body marker set attachment to shoe and the imaginary digitised marker point representing distal extremity of the shoe, **b** toe-height over time for a typical gait cycle with an MTC event and **c** without an MTC event (non-MTC gait cycle). At toe-off the toe breaks the contact with the ground and enters into the swing phase and at mx2 toe reaches the maximum vertical clearance

All participants completed informed consent procedures approved by the Victoria University Research Ethics Committee. Participants’ height, mass, age and gender were recorded at the beginning of the experiment (Table 1). A safety harness was worn while walking on the motorized treadmill. A rigid body comprising 3 infra-red emitting diodes was attached to the distal end of the right shoe and an imaginary marker was digitized at the lowest distal extremity of the shoe to represent the toe with respect to the rigid body (Fig. 1a). Three dimensional (3D) position-time coordinates of the rigid body was recorded using an Optotrak (NDI, Canada) motion tracking system at 100 Hz.

Participant’s preferred walking speed (PWS) on the treadmill was determined by first increasing the treadmill speed until the participant reported the speed to be uncomfortably fast (fast limit). It was then decreased until reported to be uncomfortably slow (slow limit). The mean of three fast and three slow limits was taken as PWS (Table 1). When required, participants were given 10–15 min familiarization before determining PWS. Participants’ comfortable walking speed while carrying a glass of water was also determined (Table 1) as above. Then they performed the following walking tasks for 5 min each (i) preferred speed walking (PW), (ii) walking while carrying a glass of water at a comfortable walking speed (dual task walking- DW), and (iii) speed-matched at DW speed without the glass of water (control walking- CW). Participants were instructed to walk without spilling water while performing the dual task walking. All participants undertook the preferred speed walking first and presentation order of the other two conditions was partially counterbalanced, such that 8 participants performed control walking followed by dual task condition, with the order reversed for 7 participants in each age group.

### Data processing

Position-time data from the Optotrak 3D motion capture system was exported to the Visual3D (C-motion, Canada) analysis software and the raw data were first interpolated to compensate any occluded signals using a window of up to 10 frames (0.1 s). A 4th order zero-lag Butterworth Filter with a cut-off frequency of 12 Hz was then applied to toe displacement data. Conditioned data were saved as text files for further processed using in-house developed MATLAB v7.2 scripts (The Mathworks, Natick, MA, USA).

MTC is found in the characteristic vertical displacement “trough” between toe-off and mx2 (Fig. 1b). To approximate toe-off, the sample frame was initially detected at which anterior-posterior toe-displacement was minimum and an 11 sample window (5 frames pre and 5 post) around this frame was established. Toe-off was then defined as the minimum vertical toe-displacement within this window. The maximum vertical displacement between successive toe-off events was then used to detect mx2. A further algorithm was devised to identify the “MTC trough” by detecting changes in the signs of the tangents of a 5-point data series comprising vertical displacement values at samples n-2, n-1, n, n + 1 and n + 2. Non-MTC gait cycles were defined as those in which a trough was not detected using the 5-point data series method (Fig. 1c). Raw position-time signals (not interpolated and not filtered) of such non-MTC gait cycles were randomly re-examined visually to ensure that non-MTC phenomenon was present in the original signal and not resulted because of any processing techniques.

A series of MTC_Heights were formed with the toe height at MTC event. MTC_Time was calculated as a percentage of total number of samples within a gait cycle using the formula:

$$ \mathrm{M}\mathrm{T}\mathrm{C}\_\mathrm{Time} = \frac{n_{MTC}}{n_{gait\  cycle}} \times 100\% $$

where *n*_*MTC*_ - number of samples from toe-off event to MTC, and *n*_*gait cycle*_ - number of total samples within the gait cycle, defined from one toe-off to the consecutive toe-off event.

Mean and standard deviation (SD) for MTC_Height and MTC_Time of all the gait cycles which had an MTC event were calculated for each individual participant in each walking condition.

Analysis of Variance (ANOVA) procedures in R package (v3.1.2) were used to analyse Age and Condition effects on the dependent variables; mean and SD of MTC_Height and MTC_Time. Two (age: young and older) X 3 (condition: PW, CW & DW) mixed model ANOVA test were performed. Condition effects were followed-up with t-tests adjusted for multiple comparisons and interaction effects were further analysed using Tukey’s post-hoc tests. In all analyses the difference between means was accepted as significant at $\alpha$ = 0.05.

For each condition total number of non-MTC gait cycles within each age group was calculated and reported as a proportion of total number of gait cycles (Table 2). A chi-square test was performed on non-MTC frequencies across walking conditions and age groups, followed by chi-square-post-hoc tests adjusted for multiple comparisons. For each subject and walking condition, mean MTC_Time was calculated using all the gait cycles which showed an MTC event. When non-MTC gait cycles were detected, the toe height at the mean MTC_Time was extracted and averaged across multiple non-MTC gait cycles for the walking condition. Averaged toe height at the mean MTC_Time were compared with respective mean MTC_Height from the same walking condition using paired t-tests.

**Table 2:** Young and older participant’s gait cycles with an MTC event and number of non-MTC gait cycles

	Young Group			Older Group		
	PW	CW	DW	PW	CW	DW
Frequency of Non-MTC gait cycles (%)^a^	2.9	26.7	22.8	18.7	34.6	37.7
Total no. of gait cycles	3759	2713	2744	4178	3261	3056
Total no. of gait cycles with MTC event	3651	1989	2119	3395	2134	1903
Total no. of non-MTC gait cycles	108	724	625	783	1127	1153
No. of participants with at least 2 non-MTC gait cycles within a trial	3	8	8	9	12	11

^a^Denotes that proportion of non-MTC gait cycles in Older Group was significantly greater than Young Group across walking conditions

## Results

**Fig. 2:**
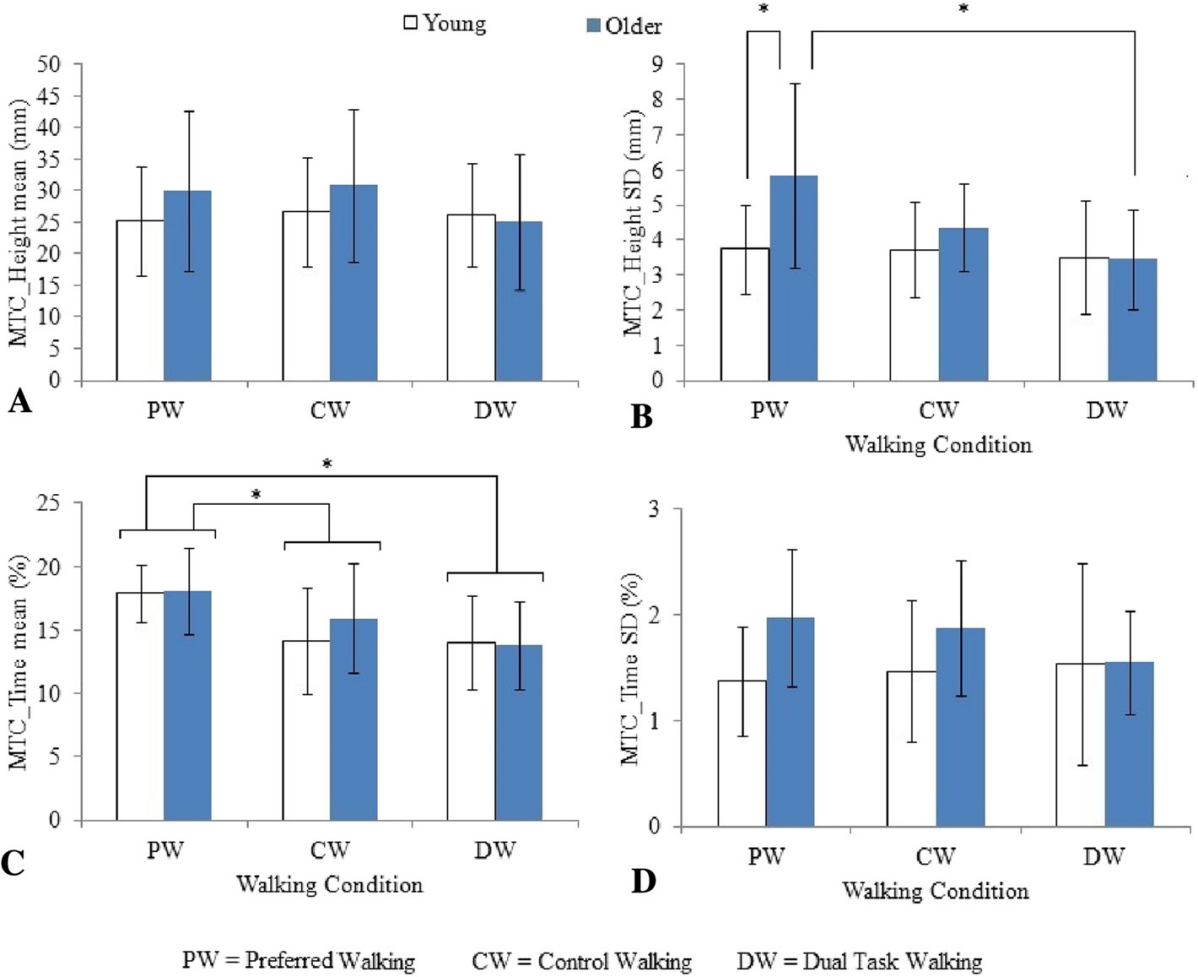
Young and older groups’ **a** mean MTC_Height, **b** MTC_Height SD, **c** mean MTC_Time, and **d** MTC_Time SD for preferred speed walking (PW), dual task walking: while holding a glass of water (DW) and matched at DW speed without a glass of water (CW). The error bars represent ± 1SD

Mean MTC_Height (Fig. 2a) did not show any effects of age (F_1,84_ = 1.428, p = 0.235), walking condition (F_2,84_ = 0.736, p = 0.482) or interactions (F_2,84_ = 0.736, p = 0.482). To exclude the possibility that non-significant results were due to low statistical power, post-hoc power analysis of mean MTC_Height was conducted (G*Power [20]) with power set at 0.80 and significance at 95 %. Results showed that samples of N = 553 would be required for the group differences between means to reach statistical significance at the .05 level. It was, therefore, improbable that the non-significant findings were attributable to sample size limitations.

MTC_Height SD (Fig. 2b) was greater in older group (F_1,84_ = 6.387, p = 0.013), and showed condition effect (F_2,84_ = 4.582, p = 0.013) and a group x condition interaction (F_2,84_ = 3.178, p = 0.047). Further, post-hoc results identified greater MTC_Height SD for the older group (5.8 mm; young – 3.7 mm) in preferred speed walking. In addition, the older participants’ MTC_Height SD in dual task walking (3.5 mm) was less than in the preferred speed baseline condition (5.8 mm). In Fig. 2c, it was interesting to note that in preferred speed walking, both groups had similar mean MTC_Time (young – 17.9 %; older – 18.0 %) despite their differences in PWS (young – 1.06 m/s; older – 0.94 m/s, p = 0.067) and stature (young – 175.1 m; older – 167.9 m, p = 0.014). ANOVA on mean MTC_Time revealed walking condition effects (F_2,84_ = 10.121, p < 10^−3^) but did not show age effects (F_1,84_ = 0.541, p = 0.464) or interactions (F_2,84_ = 0.693, p = 0.503). Post-hoc t-tests adjusted for multiple comparison showed that mean MTC_Time was shorter in both control (t_29_ = 4.42, p < 10^−3^) and dual task walking (t_29_ = 8.08, p < 10^−9^) compared to preferred gait. The final MTC parameter, MTC_Time SD, was greater in older adults than for the younger participants (F_1,84_ = 5.846, p = 0.018); there was no response to condition (F_2,84_ = 0.385, p = 0.682) and no group X condition interaction (F_2,84_ = 1.431, p = 0.245).

The highest and lowest proportions of non-MTC gait cycles were observed in the older group while dual task walking (37.7 %) and young group at preferred speed walking (2.9 %) respectively (Table 2). In preferred gait, 9 of 15 older participants demonstrated at least 2 non-MTC gait cycles, in contrast to only 3 adults in the young group. In dual task and control walking conditions, the proportions of non-MTC gait cycles and number of participants exhibiting non-MTC gait cycles increased for both groups. The overall chi-square confirmed that the non-MTC gait cycles frequencies between groups, across walking conditions were different ($\chi$^2^(2, N = 6) = 6.312, p = 0.043). Additional chi-square-post-hoc revealed that older participants demonstrated a higher frequency of non-MTC gait cycles than the young group (p = 0.039) across conditions; non-MTC gait cycle frequencies were not affected by conditions.

**Fig. 3:**
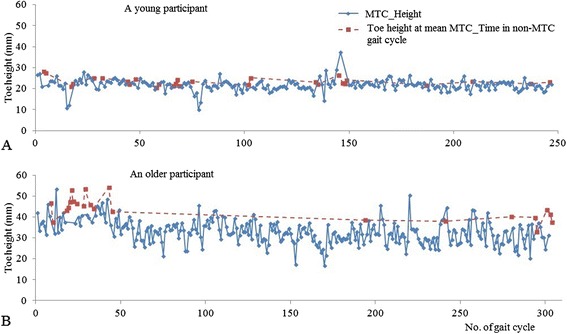
Typical time series of MTC_Height (continuous line) and extracted toe heights at the mean MTC_Time for non-MTC gait cycles (dotted line) during preferred walking for a young (**a**) and an older (**b**) participant. The number of gait cycles for the two participants differed due to self selected walking speed, cadence and stride length

**Fig. 4:**
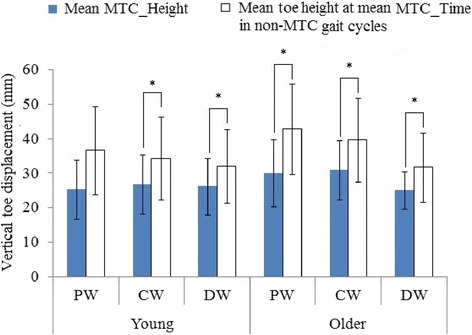
Young and older groups’ mean MTC_Height and mean extracted toe height at mean MTC_Time for non-MTC gait cycles for preferred speed walking (PW), dual task walking: while holding a glass of water (DW) and matched at DW speed without a glass of water (DW). The error bars represent ± 1SD and *denotes the significant *t*-test comparisons (p < 0.05) between mean MTC_Height and mean toe height at mean MTC_Time for non-MTC gait cycles

Figure 3 shows a typical time series of MTC_Height and extracted toe height at mean MTC_Time for non-MTC gait cycles from a young and an older participant during preferred walking condition. The figure illustrates that toe-ground clearances extracted at mean MTC_Time in non-MTC gait cycles were characteristically higher than MTC_Heights. Compared to the younger person, the older participant frequently demonstrated multiple consecutive non-MTC gait cycles. When all three conditions were combined for both groups separately, mean toe height extracted at mean MTC_Time was significantly greater than MTC_Height mean (young: t_19_ = −4.56, p < 10^−3^; older: t_31_ = −8.01, p < 10^−8^). Further, Fig. 4 presents the mean MTC_Height and mean toe height extracted at mean MTC_Time in the gait cycles which did not demonstrate an MTC event in different walking conditions for both young and older groups. For both young and older mean toe height extracted at mean MTC_Time were greater than mean MTC_Height in all the conditions, except for young in preferred walking.

## Discussion

### Age and dual task effects on MTC characteristics

Consistent with previous findings [21] in dual task walking both young and older groups reduced their walking speed. In contrast to preferred gait that showed no significant difference in speed between age groups, the older group walked significantly slower in dual task walking (0.53 m/s; young 0.42 m/s; p < 10^−3^). In the present study, therefore, speed-matched control walking condition was included to determine the divided attention effects independent of speed. Comparing the present findings with previous work is problematic because many studies of dual task effects on MTC [16] did not control walking speed. Consistent with previous studies [12–14], no age effects were found for mean MTC_Height in preferred walking condition. As hypothesised, young adults preserved MTC_Height across walking conditions but contrary to expectation older adults also maintained toe-ground clearance independent of walking task. This finding is interesting in suggesting that in response to the challenge posed by dividing attention, older pedestrians remained safe not by increasing MTC_Height as hypothesised but, as with their younger counterparts, by preserving habitual toe-ground clearance.

MTC_Height SD (variability) in preferred walking was significantly greater for the older group, usually interpreted as indicating diminished gait control [12, 13]. Most important, as hypothesized, in the older group MTC_Height SD in dual task walking (3.5 mm) was significantly lower than MTC_Height SD at preferred speed (5.8 mm). In the speed-matched control task however, MTC_Height SD was not different from preferred gait, confirming the effects of dividing attention independent of walking speed. Furthermore, the older group’s MTC_Height SD in dual task walking matched the young group’s dual task MTC_Height SD which was also the lowest of the three walking tasks. The above findings suggest that in response to increased attention demands older adults adopt a strategy of reducing MTC_Height variability but preserving MTC_Height consistent with everyday walking speed.

The present findings also uncovered lower limb control characteristics reflected in MTC timing, less frequently reported in the literature. It was hypothesised that older adults would reduce MTC_Time when dividing attention but contrary to expectation MTC_Time was not affected by age. When walking more slowly in the dual-task manipulation and speed-matched control both groups reduced MTC_Time; implying an effect of speed but not attention. This comparison emphasise the importance of speed matched control trial. Significantly higher MTC_Time variability (SD) in the older group across the walking conditions, suggested weaker MTC_Time control. In dual task walking however, older adults’ MTC_Time variability (1.6 %) approximated the young groups’ variability (1.5 %), supporting the hypothesis that it was important for the older group to precisely control MTC_Time when attention demands were high. In summary in response to increased attention demands, compared to preferred gait, the older group preserved MTC_Height but controlled the MTC event more by reducing variability in height and timing.

### An alternative gait control strategy – non-MTC gait cycles

Non-MTC gait cycles were relatively frequent but they have not been commonly reported in previous investigations of toe-ground trajectory control during walking. In non-MTC gait cycles changes to toe trajectory eliminated the MTC event and, as hypothesized, statistical analysis revealed that non_MTC gait cycles were significantly more frequent in older adults independent of walking task. While non_MTC frequencies chi-square analysis did not indicate significant condition effects, non-MTC gait cycles in young people were generally less common in preferred walking but when their gait was challenged or de-stabilized*,* in the present study by carrying a glass of water or walking slowly, more non-MTC gait cycles (Table 2) appeared. Further, the number of younger participants exhibiting at least 2 non-MTC gait cycles, increased from 3 of 15 in preferred walking to 8 in both dual task and control conditions. In contrast, 9 of 15 older participants exhibited non-MTC gait cycles even at preferred gait. Furthermore, in such non-MTC gait cycles, toe-height at mean MTC_Time exceeded mean MTC_Height, suggesting that in non-MTC strides, toe-ground clearance at mid-swing (the usual MTC_Time) is maintained higher. The findings presented here are the first to propose that eliminating the biomechanically critical MTC event, i.e. adopting non-MTC gait cycles, is a locomotor control strategy that may be adaptive in reducing the likelihood of toe-ground contact when gait is challenged.

In determining the “adaptive” characteristics of toe height control at MTC, a limitation of the present study is that it was conducted only in treadmill walking. The reported age and attentions effects on the central tendency and variability of MTC_Height and MTC_Time should be confirmed in overground walking, furthermore, the frequency of non-MTC gait cycles may be different in overground walking. A further limitation of the present study was that the ageing and task effects on MTC was limited to mean and standard deviation measures, therefore, an interesting dimension to the future studies would be the characterization of other MTC distribution parameters such as skewness, kurtosis, median, IQR, 1st quartile, 3rd quartile and maximum-minimum range [12]. In addition, probability modelling [12] was not conducted to specify precisely the “risk” of toe-ground contact across the walking conditions. MTC_Height distribution analysis, including skewness, central tendency and variability could be employed to determine whether the reported MTC control adaptations to dual task walking significantly reduce tripping risk. While cross-sectional data are generally acceptable for characterising age effects on gait biomechanics, longitudinal designs should also be employed to confirm “ageing” effects on gait variables.

## Conclusion

This study showed that older adults adopt a strategy of preserving MTC_Height and precisely controlling MTC by reducing both height and timing variability in response to increased attention demands. The present study is the first to investigate non-MTC gait cycles as a biomechanical parameter to characterize age and divided attention effects on lower limb trajectory control. It is proposed that an adaptive biomechanical strategy to attain higher mid-swing clearance is to eliminate the critical MTC event, increasing the frequency of non-MTC gaits cycles. Future work could determine the association between lower limb joint angles and non-MTC gait cycles. Furthermore, MTC_Time and the proportion of non-MTC gait cycles could be investigated in populations with lower limb control impairments associated with very high risk of tripping-related falls, such as stroke and neurological disease.

## Abbreviations

MTC: Minimum toe clearance
3D: Three dimensional
yr: Year
PW: Preferred walking
DW: Dual task walking
CW: Control walking
SD: Standard deviation
ANOVA: Analysis of variance
